# The relationship between plasma amino acids and circulating albumin and haemoglobin in postabsorptive stroke patients

**DOI:** 10.1371/journal.pone.0219756

**Published:** 2019-08-14

**Authors:** Roberto Aquilani, Roberto Maestri, Mirella Boselli, Maria Pia Achilli, Nadia Arrigoni, Mariella Bruni, Maurizia Dossena, Manuela Verri, Daniela Buonocore, Evasio Pasini, Annalisa Barbieri, Federica Boschi

**Affiliations:** 1 Dipartimento di Biologia e Biotecnologie Università degli Studi di Pavia, Italy; 2 Istituti Clinici Scientifici Maugeri IRCCS, Servizio di Bioingegneria, Istituto di Montescano, Montescano, Pavia, Italy; 3 Istituti Clinici Scientifici Maugeri IRCCS, Unita di Riabilitazione Neuromotoria, Unita Gravi Cerebrolesioni Acquisite, Istituto di Montescano, Montescano, Pavia, Italy; 4 Istituti Clinici Scientifici Maugeri IRCCS, Servizio di Patologia Clinica, Istituto di Pavia, Pavia, Italy; 5 Istituti Clinici Scientifici Maugeri, IRCCS, Divisione di Riabilitazione Cardiologica, Istituto di Lumezzane, Lumezzane, Bergamo, Italy; 6 Dipartimento di Scienze del Farmaco, Università degli Studi di Pavia, Italy; Nathan S Kline Institute, UNITED STATES

## Abstract

**Background:**

This retrospective study had two main aims: (1) to document possible correlations between plasma Amino Acids (AAs) and circulating Albumin (Alb) and Haemoglobin (Hb); and (2) to identify which AAs were predictors of Alb and Hb.

**Methods:**

The study considered 125 stroke subjects (ST) (61.6% males; 65.6 +/- 14.9 years) who met the eligibility criteria (absence of co morbidities associated with altered plasma AAs and presence of plasma AAs determined after overnight fasting). Fifteen matched healthy subjects with measured plasma AAs served as controls.

**Results:**

The best correlations of Alb were with tryptophan (Trp) and histidine (His) (r = + 0.53; p < 0.0001), and those of Hb were with histidine (r = +0.47) and Essential AAs (r = +0.47) (both p<0.0001). In multivariate analysis, Trp (p< 0.0001) and His (p = 0.01) were shown to be the best positive predictors of Alb, whereas glutamine (p = 0.006) was the best positive predictor of Hb.

**Conclusions:**

The study shows that the majority of plasma AAs were positively correlated with Alb and Hb. The best predictors of circulating Alb and Hb were the levels of tryptophan and glutamine, respectively.

## Introduction

Stroke is an important cause of disability and the third cause of death in Western Societies [1,2]. In Italy, about one third of stroke survivors are totally dependent on other people one year after a stroke [3]. The first 30 days after the acute event are crucial for both patient survival and functional prognosis, as 20% of mortality and 80% of neurological recovery occur in this time [4,5].

It is therefore essential to make every effort to achieve the best rehabilitative outcomes during the first month after a stroke.

Considering the lack of availability of drugs to repair the brain area damaged by vascular accidents, nutritional interventions seem to be a promising tool to enhance post-stroke neurocognitive retrieval. Nutrition appears to influence the repair process of damaged brain tissue. An observational study demonstrated that spontaneous neurocognitive recovery in stroke subjects was associated with protein-calorie intakes [6]. Moreover, when stroke patients were supplemented with proteins [7], zinc [8] and Essential Amino Acids (EAAs) [9], their neurocognitive retrieval improved.

Improving circulating Albumin (Alb) and Haemoglobin (Hb) levels may be a way of influencing neurocognitive recovery.

Alb therapy has been shown to be neuroprotective in animal models of transient focal ischemia [10–12], global ischemia [13] and traumatic brain injury[14].

In subjects with subacute stroke (<3 months from the acute event), the gain in serum Alb correlated with the gain in functional independence [15].

With respect to Hb, the circulating protein levels were significantly linked to the functional recovery of stroke subjects during the rehabilitation phase of the disease [16].

The crucial problem, however, is to determine whether and how is it possible to increase Alb and Hb levels in subacute stroke subjects, i.e. in a phase of the disease during which systemic inflammation, even though it is declining, may persist [16], and to maintain alterations of the two circulating protein levels [17]. It is not feasible to act on the extra vascular redistribution and degradation rate of Alb in order to increase its circulating levels, given the scarce information about these processes. In addition, providing patients with Alb as a nutrition support agent is not appropriate [18]. In subjects with mild anaemia, it is not appropriate to administer transfusion or erythropoietin.

At present, the only potential way of increasing Alb and Hb levels is to use nutritional interventions to increase their synthesis. In healthy individuals, Alb synthesis is acutely stimulated after ingestion of a complete meal [19–21] or of carbohydrates and fats without protein [21]. The ingestion of protein alone is equally as effective as the ingestion of a complete meal in stimulating Alb synthesis [22]. This indicates that Amino Acids (AAs) play an important role in regulating Alb synthesis. Indeed, AAs influence Alb synthesis both directly and indirectly [23–27] by stimulating the release of insulin [21]. A third of the AAs in a daily dietary intake are used in the synthesis of Alb and other plasma proteins [28]. In inflamed elderly subjects who had been operated for hip fractures, supplemented EAAs increased circulating Alb and Hb levels [17]. Early in vitro and in vivo experiments had previously documented the importance of AAs in stimulating liver Alb (and other protein) synthesis [24–26, 29].

In addition, Hb synthesis is sensitive to AA availability as these substrates influence erythropoietin production [30]. Amino aciduria negatively correlated with plasma Hb levels [31]. More recently, a study reported that Hb synthesis in chickens is influenced by energy intake [32].

On the basis of these studies, we wanted to develop our understanding of the relationship between circulating AAs and circulating Alb/Hb levels in subacute stroke subjects under overnight fasting conditions instead of after meal stimulation. Therefore, this retrospective observational study had two aims: to document any associations between circulating AAs and circulating Alb and Hb and to highlight the best AA predictors of Alb and Hb.

To this end, we formulated two hypotheses. Firstly, that plasma AAs and circulating Alb/Hb may be positively associated given that (a) during overnight fasting, the synthesis of Alb continues even though at a reduced rate [33]; (b) during overnight fasting, the liver is continuously exposed to circulating AAs whose availability is important for Alb synthesis [22,24]; (c) AA availability is essential to haemopoiesis [30]. AA deficiency reduces protein synthesis in a number of cell types including reticulocytes [34].

The second hypothesis we put forward was that plasma AA Tryptophan (Trp) and isoleucine could predict circulating Alb and Hb levels, respectively. This hypothesis relied on previous studies, which had documented that Alb synthesis in fasting rabbits [35] was stimulated by Trp and, to a lesser extent, isoleucine, but not by a mixture of other AAs. Isoleucine was essential for haemopoiesis in experimental anaemic rats [36].

If we were correct, the findings of the study would provide a useful framework for future investigations into how to increase Alb/Hb during short fasting periods in stroke subjects who often have insufficient nutrition intakes [37,38].

## Subjects and methods

### Population

This is a retrospective study carried out on stroke patients who were admitted to our Rehabilitation Centre (Rehab) from January 2004 to January 2014.

The patients were selected from our database and were deemed eligible if (1) admission occurred within 35 days after the acute event; (2) they were not affected by diseases that were potentially associated with alterations in plasma amino acids (chronic heart failure, cancer, chronic obstructive pulmonary disease, steroid therapy, diabetes) or by diseases that led to hypoalbuminemia/anaemia (albumin losses from nephrotic syndrome, ascitis, protein–losing enteropathy, overdegradation following trauma or surgery, but not neurosurgery due to stroke). Moreover, patients were only included if their plasma AAs, anthropometrics and routine biohumoral variables had been measured, their nutritional intakes had been analysed, and if there was computerised tomography documentation of vascular brain damage.

Out of a total of 236 subjects in the database, 125 (approximately 53% patients) were selected for this study. The patients came from neurosurgery settings (12%), stroke units (36%) and neurological wards (52%). Computerised tomography documented that 60.8% had brain ischemic damage and 39.2% had brain haemorrhage damage. On admission to Rehab, 58.4% (n = 73) were fed via percutaneous endoscopy gastrostomy (PEG) and 41.6% (n = 52) were self-feeding.

### Description and analysed variables

#### Anthropometric characteristics

Body weight (BW) (kg) had been measured using a mechanical weight lifter; height (m) had been calculated from knee height [39].
Body Mass Index (BMI) was calculated as kg x m^-2^.
For this study a loss of actual BW in relation to habitual (pre-event) BW>5% (i.e. actual/habitual BW<95%) was considered as an index of significant undernutrition [40].

#### Biohumoral variables

- Routine variables, including serum protein electrophoresis.
- Markers of body inflammation:
  - C-Reactive Protein (CRP, normal value<0.3 mg dl^-1^) determined by an immune-turbidimetric method.
  - Acute phase reactant proteins (fibrinogen, normal values 350–495 mg / dl; α_1_-globulin system, normal values 210–350 mg dl^-1^) and non-reactant proteins (albumin, normal values 4.02–4.76 g/dl; prealbumin, normal values 18–30 mg/dl; transferrin, normal values 202–364 mg/dl).

#### Plasma amino acids

The following protocol had been used to measure plasma AA levels. At 8:am after overnight fasting, the patients underwent venipuncture from a basilic vein in the unaffected arm to sample blood to determine the concentrations of plasma AAs. Concentrations of free AAs in the plasma were measured using an Amino Quant II amino acid analyser, based on the HP 1090 HPLC system, with fully automated precolumn derivatisation, using both orthophthal-aldehyde and 9-fluorenyl-methyl-chloformate reaction chemistries according to the manufacturer’s protocol.

The measurements of the plasma amino acids had been carried out in triplicate by the same laboratory.

The mean of the three measurements had been calculated and adopted. The characteristics of the method were based both on precision and standardisation properties:

- Precision (Relative Standard Deviation (RSD) was 1.13%
- Reliability (bias) was 10.37%
- The lower limit of quantitation was 0.18ng/ml
- The limit of detection was 0.6ng/ml

For the measurements in triplicate, the intra-day variability (RSD) was 3.21% and the inter-variability was 4.17%.

The results were obtained by injecting 1μL of the derivatised mixture and measuring absorbance simultaneously at 338 and 262nm. Plasma concentrations were expressed as μmol/L.
As a comparison we considered the amino acid measurements carried out in 15 healthy sedentary subjects (no history of disease, no clinical or instrumental signs of disease) during the period 2004–2014 with a similar age (69.3±4.7 years), sex distribution (60% female) and body mass index (23.1±3.6 Kg/m^2^). The venous blood samples had been drawn at 8:am, after overnight fasting.

#### Nutritional intakes

These had been measured following our Standard Protocol [40].
In brief, for self-feeding patients (n = 52) a 3-day food diary was kept by the rehabilitation nurses, who had previously been taught how to do so. The nurses recorded the type and weight of cooked or uncooked food selected by patients from the hospital catering menu on a diet sheet for 3 days both before and after the patients’ meals. The amount of food that was actually ingested was converted to its raw equivalent when necessary, using appropriate tables [41].
For this study, we re-analysed the nutritional intakes using a computer Program Food Database DR3 (Dieta ragionata 3. Sintesi informatica, University of Pavia, Italy), in order to calculate ingested calories and macro-/micronutrients.
Nutritional intakes from the pharmaceutical formula given to patients on enteral nutrition (n = 73 of whom 51.6% were dysphagic) were calculated from the nutritional composition reported in the formula labels.
Daily calories and protein intakes were considered adequate to body requirements if they were ≥25 Kcal/Kg and, respectively ≥1g/Kg [42].

#### Functional status

Evaluated using the functional independence measure (FIM) [15].

The study was approved by the local Institutional Review Board and by the Istituti Clinici Scientifici Maugeri Ethics Committee (approval date 16/05/2017, protocol number 2113 CE). All patients or, where the case, their relatives or appointed caregivers, had signed an informed written consent prior to the participation to the study and provided written consent for the scientific treatment of their data in an anonymous form. The decision whether a patient had capacity to consent (i.e. the ability to use and understand information to make a decision and communicate any decision made) or consent had to be given by relatives or appointed caregivers was based on judgement by appropriately trained and experienced health professionals.

## Statistical analysis

Descriptive statistics of all collected data were reported as mean ± SD for continuous variables and as percentage frequency for categorical variables. The Shapiro–Wilk statistic supported by visual inspection was used to test the normality of the distribution of all variables.

Between-group comparisons for continuous variables were carried out by the unpaired Student T-test or by the Mann–Whitney U-test, if appropriate. The Chi-squared test was used for categorical variables.

The association between circulating Alb and Hb levels and plasma AAs was assessed by the linear Pearson correlation coefficient (Pearson r) or by the Spearman's rank correlation coefficient (Spearman r), as appropriate.

Multivariable linear regression analysis was used to assess the association between AAs, considered as explanatory variables, and Albumin and Haemoglobin (dependent variables), respectively, in stroke patients.

A p-value of <0.05 was considered statistically significant. When appropriate, false discovery rate (FDR) was controlled at 5% using the Benjamini-Hochberg method and FDR adjusted p-values were also reported. [43]. All analyses were carried out using the SAS/STAT statistical package, release 9.4 (SAS Institute Inc., Cary, NC, U.S.A.).

## Results

### Patient characteristics

Table 1 reports the baseline characteristics of the stroke population at admission to the Rehabilitation Institute. The patients were slightly overweight, with an average of 3% loss of habitual (pre-event) body weight (BW), and systemic inflammatory state (increased values of C-Reactive Protein (CRP) and Erytrocyte Sedimentation rate (ESR). Calorie-protein intakes were lower than recommended. Serum total protein levels were normal but Alb concentration was lower than normal values. 69 subjects (55.2%) had Alb<3.5 g/dl. Anaemia was present in 42 patients (33.6%). The entire patient population displayed severe physical disability (Functional Independence Measure (FIM) -52.4% of normal values).

**Table 1:** Demographic, clinical, anthropometric, biohumoral, functional and nutritional variables of the analysed stroke population in the study.

Variables	Stroke population (n = 125)	Normal values	%RDA
**Demographic**			
Male/Female	77/48	-	
Age (years)	65.6±14.9	-	
**Clinical**			
Aetiology:			
Ischemic	82 (65.6%)	-	
Haemorrhagic	43 (34.4%)	-	
**Anthropometric**			
Actual body weight (kg)	71.96±15.38	-	
Body mass index (BMI) (Kg/m^2^)	25.62±4.56		
Pre-event body weight (kg)	74.91±15.89		
Actual/pre-event BW (%)	97±7	-	
**Blood**			
ESR 1^st^ hr (mm)	37±30.25	<20	
Haemoglobin (g/dl)	13±1.93	12–15	
Blood urea (mg/dl)	40.38±20.88	20–40	
Serum creatinine (mg/dl)	0.97±0.28	0.7–1.2	
Plasma glucose (mg/dl)	110±36.5	80–110	
α_1_ globulin (g/dl)	0.22±0.05	0.21–0.35	
Fibrinogen (mg/dl)	389.67±97.34	350–500	
Serum albumin (g/dl)	3.34±0.56	4.02–4.76	
Serum prealbumin (mg/dl)	21.1±5.8	18–30	
Serum transferrin (mg/dl)	204.7±48.38	202–364	
Serum C-reactive protein (CRP) (mg/dl)	1.38±2.44	<0.3	
Total proteins (g/dl)	7.09±0.63	6.2–8	
White cell counts (n°/mm^3^)	6669.28±1957.01	4000–9000	
Neutrophils (%)	60±10.25	45–75	
Lymphocytes (%)	27.54±9.54	20–47	
**Function**			
FIM (score)	59.49±28.03	126	
**Daily nutritional intake**			**%RDA**
Energy			
Kcal/d	1561±186	-	-
Kcal/Kg	21.8±2.01	≥25	87.2
Proteins			
g/d	66.2±11.7	-	-
g/Kg	0.92±0.16	≥1.0	92
Lipids			
g/d	52.4±10.1	-	-
g/Kg	0.73±0.14	≤1	100
Carbohydrates			
g/d	206±45.1		
g/Kg	2.86±0.57	-	-
Calcium (mg)	749±90	1000	74.9
Phosphorus (mg)	1324±177	1000	132.4
Potassium (mg)	3650±767	3100	118
Sodium (mg)	1541±139	-	-
Iron (mg)	16.1±3.2	10	161
Copper (mg)	1.46±0.58	1.2	121.7
Thiamin (mg)	1.2±0.3	1.1	109
Riboflavin (mg)	1.83±0.4	1.1	166
Niacin (mg)	19.1±7.6	16	119
Vitamin C (mg)	159.5±68.8	60	266
Folic Acid (μg)	171±38	400	42.8
Vitamin B_12_ (μg)	2.1±0.85	2.4	87.5

Data are expressed as mean ±standard deviation (SD)

FIM = functional independence measure; ESR: erythrocyte sedimentation rate; RDA: recommended daily allowance; nd: not defined

Compared to the healthy controls, the stroke subjects showed profound alterations of plasma AA concentrations (Table 2), with 71.4% of AAs higher than in healthy controls histidine (His), serine, citrulline, ornithine, glycin, threonine, alanine, phenylalanine, tyrosine, methionine, lysine and Branched Chain AAs (BCAAs: leucine, valine, isoleucine). In contrast, aspartic acid, glutamic acid, asparagine and taurine concentrations were lower in stroke subjects than in healthy controls.

**Table 2:** Differences in plasma Amino Acid concentrations (μmol/L) and Amino Acid ratios between stroke patients and healthy subjects.

Variables	Healthy subjects (n = 15)	Stroke patients (n = 125)	p value	FDR-adjusted p value
Aspartic Acid	60.42±52.11	11.38±2.26	0.053	0.0622
Glutamic Acid	157.90±55.61	71.78±23.53	<0.0001	<0.0001
Histidine	53.71±11.05	72.02±15.45	0.0001	0.0002
Asparagine:	54.43±11.87	49.14±11.91	0.033	0.0405
Serine	68.77±23.30	113.46±29.36	<0.0001	<0.0001
Glutamine	570±70	576.45±116.81	0.8	0.8
Arginine	60.49±9.62	59.27±19.21	0.40	0.45
Citrulline	24.57±3.66	32.83±9.78	0.009	0.0128
Glycine	208.37±76.81	269.35±68.01	0.023	0.0296
Threonine	104.39±22.60	137.96±41.78	0.001	0.0018
Alanine	284.41±51.22	345.04±92.03	0.009	0.0128
Taurine	133.25±23.03	94.68±29.73	0.0008	0.0015
Tyrosine	49.40±10.66	65.08±17.50	0.0005	0.0010
Valine	152.62±19.59	247.77±68.66	<0.0001	<0.0001
Methionine	15.17±7.42	28.58±8.09	<0.0001	<0.0001
Tryptophan	43.31±11.19	42.44±11.56	0.53	0.5504
Phenylalanine	41.61±7.28	61.69±16.43	<0.0001	<0.0001
Leucine	78.52±13.69	136.15±38.38	<0.0001	<0.0001
Ornithine	56.38±6.39	87.29±31.16	0.002	0.0034
Lysine	103.41±24.95	217.77±55.76	<0.0001	<0.0001
Isoleucine	44.15±7.45	71.06±18.29	<0.0001	<0.0001
Total Amino Acids (TAAs)	2791±404.8	2574.55±404.80	<0.0001	<0.0001
Branched Chain Amino Acids (BCAAs)	275.28±37.72	454.98±117.74	<0.0001	<0.0001
Essential Amino Acids (EAAs)	583.17±74.78	941.55±191.13	<0.0001	<0.0001
EAAs /TAAs (%)	32±6	33.7±3.2	0.003	0.0048
BCAAs /TAAs (%)	15±4	16.3±2.7	0.020	0.0270
BCAAs /EAAs (%)	47±4	48±5	0.46	0.50

Data are expressed as mean ±standard deviation (SD)

p-values are taken from the Mann-Whitney U test; FDR-adjusted p value: p values for false discovery rate controlled at 5% using the Benjamini-Hochberg method

The only AAs that were similar in patients and controls were Trp, arginine and glutamine (Gln).

As a consequence of AA perturbations, plasma levels of Total AAs (TAAs), EAAs, BCAAs in addition to EAA/TAA and BCAA/TAA ratios were higher in stroke subjects than in controls.

### Relationships between plasma AA and circulating Alb/Hb

Table 3 reports the correlations between plasma AAs and circulating Alb/Hb in post absorptive stroke subjects.

**Table 3:** Correlations between circulating albumin and haemoglobin levels and plasma amino acid concentrations in post absorptive patients.

Variables	Albumin	P value	FDR-adjusted p value	Variables	Haemoglobin	P value	FDR-adjusted p value
Tryptophan	0.533	<0.0001	<0.0001	Histidine	0.47	<0.0001	<0.0001
Histidine	0.53	<0.0001	<0.0001	Essential Amino Acids (EAAs)	0.47	<0.0001	<0.0001
Total Amino Acids (TAAs)	0.33	0.0002	0.0011	Branched Chain Amino Acids (BCAAs)	0.42	<0.0001	<0.0001
Serine	0.31	0.0004	0.0015	Total Amino Acids (TAAs)	0.39	<0.0001	<0.0001
Alanine	0.31	0.0004	0.0015	Leucine	0.36	<0.0001	<0.0001
Essential Amino Acids (EAAs)	0.29	0.0009	0.0028	0.34	<0.0001	<0.0001	<0.0001
Glutamine	0.27	0.003	0.0083	Valine	0.32	0.0002	0.0006
Leucine	0.26	<0.0001	0.0001	Lysine	0.32	0.0002	0.0006
Asparagine	0.24	0.01	0.015	Glutamine	0.29	0.0009	0.0022
Threonine	0.24	0.006	0.013	Alanine	0.26	0.003	0.0066
Valine	0.24	0.008	0.015	Cytrulline	0.23	0.009	0.018
Lysine	0.24	0.006	0.013	Ornithine	0.22	0.01	0.018
Branched Chain Amino Acids (BCAAs)	0.24	0.008	0.015	Serine	0.21	0.02	0.034
Arginine	0.22	0.01	0.015	Aspartic Acid	0.19	0.03	0.044
Cytrulline	0.22	0.01	0.015	Glutamic Acid	0.19	0.03	0.044
Isoleucine	0.18	0.04	0.055	Asparagine	0.18	0.05	0.065
Aspartic Acid	0.15	0.09	0.14	Threonine	0.17	0.06	0.07
Methionine	0.15	0.1	0.12	Tyrosine	0.17	0.06	0.07
Ornithine	0.14	0.12	0.14	Methionine	0.17	0.05	0.06
Tyrosine	0.11	0.21	0.23	Glycine	0.16	0.07	0.08
Glutamic Acid	0.09	0.3	0.31	Arginine	0.05	0.6	0.63
Phenylalanine	-0.08	0.36	0.3600	Phenylalanine	-0.01	0.9	0.9

Data are expressed as mean ±standard deviation (SD)

p-values are from the Mann-Whitney U test; FDR-adjusted p value: p values for false discovery rate controlled at 5% using the Benjamini-Hochberg method

**Fig 1:**
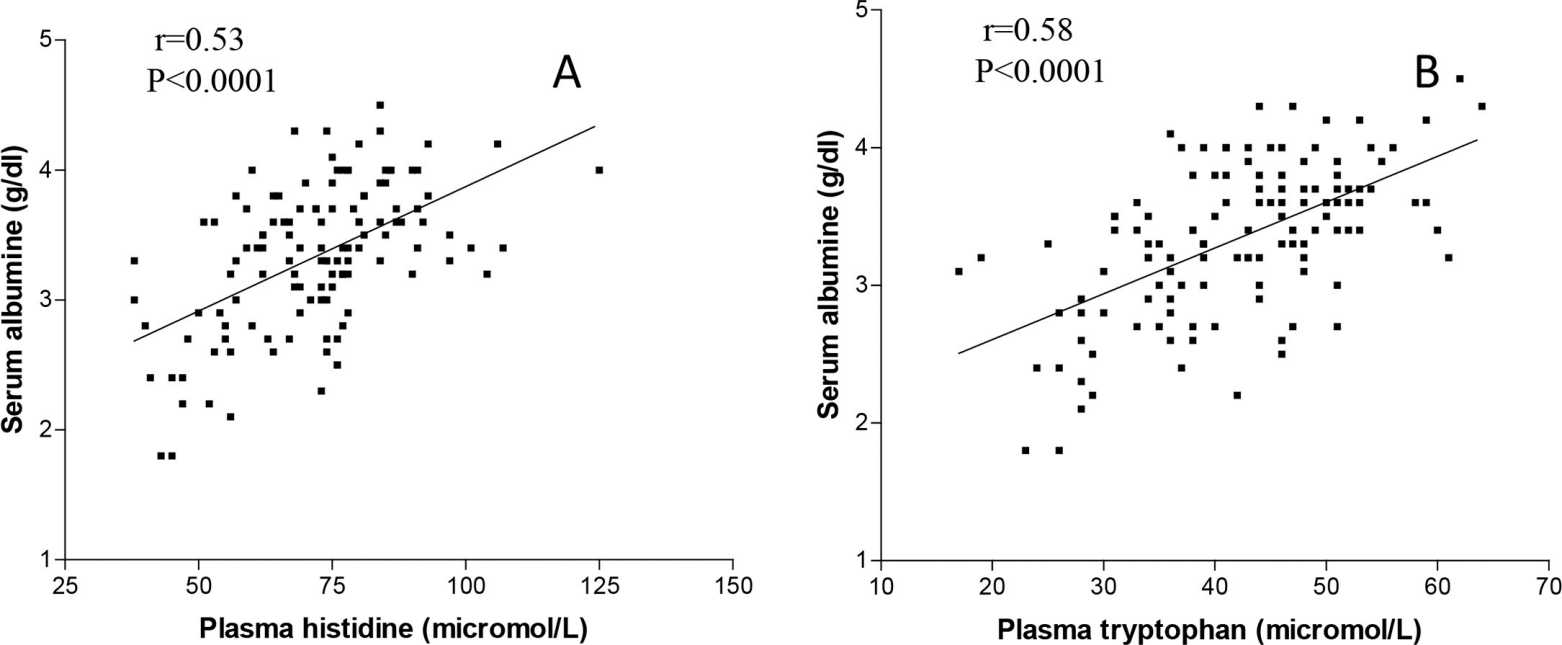
Correlations between plasma tryptophan (panel A), plasma histidine (panel B) and serum albumin.

Alb was positively, significantly correlated with all the measured AAs except for aspartic acid, methionine, ornhitine, tyrosine, glutamic acid and phenylalanine. The most important correlations of Alb were with Trp (r = +0.533, p<0.0001) and His (r = +0.53, p<0.0001) (Fig 1 panel A and B). The magnitude of the correlations increased when the two AAs were combined (r = +0.60, p<0.0001).

The correlation between Trp and Alb remained significant even when the levels of Alb were normalised for the levels of CRP (Alb/CRP) (r = +0.426, p<0.0001).

**Fig 2:**
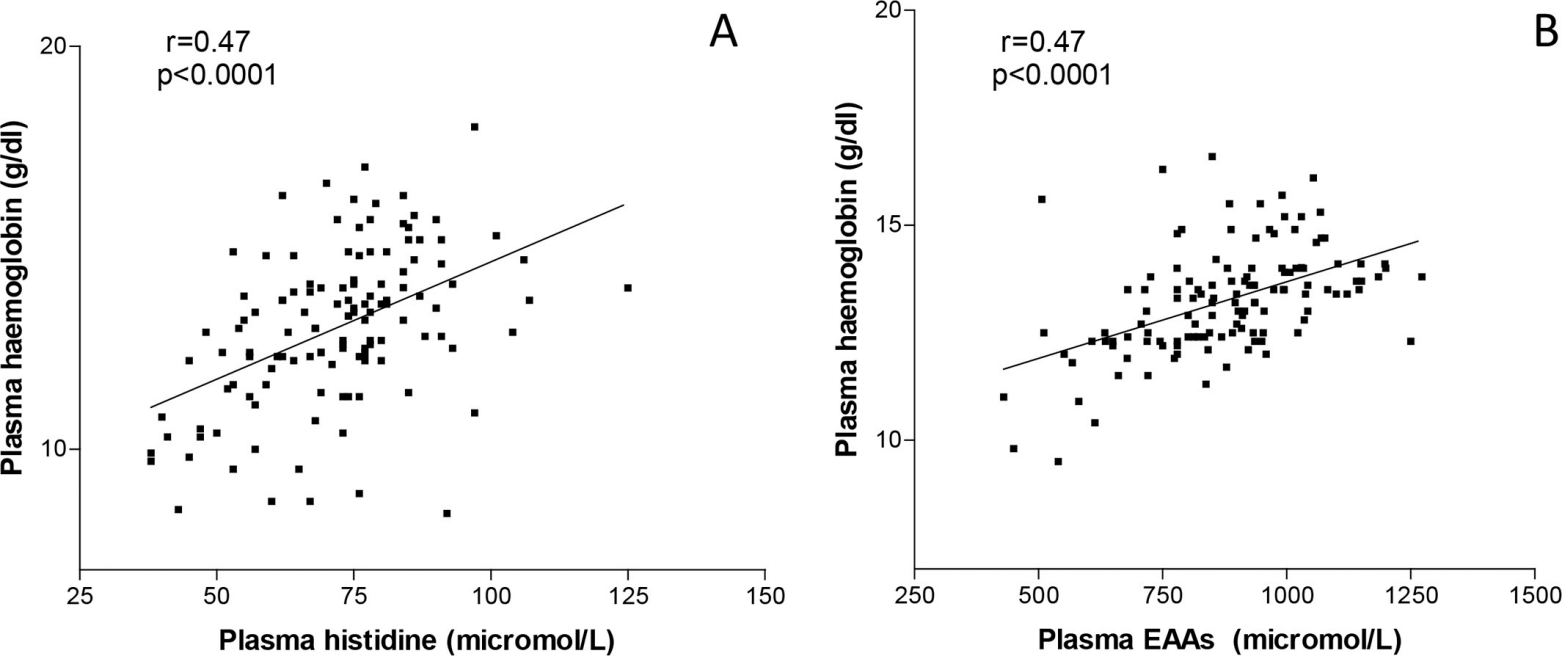
Correlations between plasma histidine (panel A), plasma essential amino acids (panel B) and plasma haemoglobin.

Circulating Hb was positively, significantly associated with 77.4% of plasma AAs and was not correlated with Threonine, tyrosine, glycin, arginine or phenylalanine. The best correlations were with His (r = +0.47, p<0.0001) and EAAs (r = +0.42, p<0.0001) (Fig 2 panel A and B).

In multivariate analysis (Table 4), the AA Trp, His (positive association) and phenylalanine (inverse association) were found to be independent predictors of Alb (p<0.0001, p = 0.016 and p = 0.0001, respectively) while Glutamine (p = 0.006, direct association), asparagine (p = 0.006, negative association), Glycine (p = 0.009, negative association) and Aspartic acid (p = 0.03, positive association) were found to be the only independent predictors of Hb.

**Table 4:** Multivariate analysis showing the best amino acid positive and negative predictors of albumin and respectively haemoglobin in the stroke patients.

Variables	Albumin t value	p value	Variables	Haemoglobin t value	p value
Tryptophan	5.08	<0.0001	Glutamine	2.8	0.006
Histidine	2.46	0.01	Asparagine	-2.81	0.006
Phenylalanine	-4.01	0.0001	Glycine	-2.68	0.009
			Aspartic Acid	2.23	0.03

To highlight the distributions of patient characteristics in relation to the most relevant positive predictors, we stratified the population for Trp≤35μmol/L (Trp ≤35) and Trp >35μmol/L (Trp >35) and respectively for Gln≤500 μmol/L (Gln ≤500) and Gln>500 μmol/L (Gln >500), Trp = 35 and Gln = 500 being thresholds obtained by rounding the values of the lower quartiles.

Table 5 reports the distribution of circulating Alb and Hb in relation to the Trp and respectively Gln cut-off points.

**Table 5:** Distribution of normoalbuminemic and hypoalbuminemic stroke patients in relation to plasma tryptophan (Trp) levels, and of anaemic patients in relation to plasma glutamine (Gln) concentrations. The concentrations of the amino acids are expressed as μmol/L.

	Albumin ≥3.5	Albumin <3.5
*Tryptophan >35	51 (92.7%)	42 (60.9%)
Tryptophan ≤35	4 (7.3%)	27 (39.1)
	No anaemia	Anaemia
°Glutamine >500	68 (81.9%)	29 (69%)
Glutamine ≤500	15 (18.1%)	13 (31%)

* χ 2 test p<0.0001

°χ 2 test: p = 0.1

After stratification (Table 6), subjects with Trp ≤35 (25% of the population), in comparison to subjects with Trp >35, showed significant loss (7%; p = 0.002) of pre-event body weight, lower protein-energy intakes, lower serum levels of negative proteins of the acute phase response (Alb, preralbumin, transferrin) but a higher inflammatory state (CRP, p = 0.002; α_1_ globulin, p = 0.004) and physical disability.

**Table 6:** Differences in anthropometric, biohumoral, functional status and nutritional intakes of stroke patients after stratifications in the groups with plasma tryptophan levels >35 and respectively ≤35 μmol/L.

Variables	Tryptophan >35 (n = 94)	Tryptophan ≤ 35 (n = 31)	p value	FDR-adjusted p value
**Anthropometric**				
Body mass index (BMI) (Kg/m^2^)	26.02±4.30	24.42±5.17	0.09	0.15
Actual/pre-event BW (%)	98±0.6	93±0.1	0.002	0.007
**Blood**				
ESR 1^st^ hr (mm)	32.05±26.13	52±36.85	0.001	0.007
Haemoglobin (g/dl)	13.32±1.81	12.10±2.04	0.002	0.007
Blood urea (mg/dl)	40.05±18.62	41.35±26.96	0.76	0.80
Serum creatinine (mg/dl)	0.98±0.27	0.95±0.33	0.70	0.80
Plasma glucose (mg/dl)	109.85±36.35	110.26±37.32	0.96	0.9600
α_1_ globulin (g/dl)	0.21±0.05	0.24±0.05	0.004	0.012
Fibrinogen (mg/dl)	383.25±93.91	408.28±106.19	0.23	0.35
Serum albumin (g/dl)	3.49±0.48	2.88±0.51	<0.0001	0.0002
Serum prealbumin (mg/dl)	21.77±5.47	19±6.26	0.022	0.046
Serum transferrin (mg/dl)	213.73±47.39	178.17±41.57	0.0004	0.004
Serum C-reactive protein (CRP) (mg/dl)	0.99±1.47	2.54±3.99	0.002	0.007
White cell counts (mm^3^)	6732.77±1951.1	6476.77±1994.25	0.53	0.70
Neutrophils (%)	59.51±9.96	61.55±11.10	0.34	0.48
Lymphocytes (%)	28.26±9.29	25.36±10.09	0.74	0.80
**Function**				
FIM (score)	62.85±28.30	49.42±24.98	0.020	0.046
**Daily nutritional intake**				
Energy				
Kcal/d	1760±275			
Kcal/Kg	23.9±3.72	19.5±2.05	<0.05	0.076
Proteins				
g/d	78.2±12.1	54.5±9.8		
g/Kg	1.06±0.15	0.78±0.13	<0.02	0.026
Lipids				
g/d	53.9±15.9			
g/Kg	0.8±0.22	0.65±0.09	0.6	0.74
Carbohydrates				
g/d	228.2±56.7	183.9±33.5		
g/Kg	3.08±1.1	2.64±0.14	0.07	0.12

Data are expressed as mean ±standard deviation (SD)

p-values are from the unpaired Student t-test; FDR-adjusted p value: p values for false discovery rate controlled at 5% using the Benjamini-Hochberg method

FIM = functional independence measure; ESR: erythrocyte sedimentation rate

Subjects with Gln ≤500 (Table 7, 22.4% of the population), compared to those with Gln >500 displayed lower Hb, Alb and transferrin concentrations but a higher inflammatory state (high CRP, ESR, α1 globulin, fibrinogen, white cell counts, neutrophils %). Lymphocytes % were lower in the Gln<500 group (p = 0.04). The rates of lost pre-event body weight (in 22.4% of the patients) and nutrient intakes (Table 7A) did not vary between the two groups.

**Table 7:** Differences in anthropometric, biohumoral, functional status and nutritional intakes of stroke patients after stratifications in the groups with plasma glutamine levels >500 and respectively ≤500 μmol/L.

Variables	Glutamine >500 (n = 97)	Glutamine ≤ 500 (n = 28)	p value	FDR-adjusted p value
**Anthropometric**				
Body mass index (BMI) (Kg/m^2^)	25.62±4.42	25.62±5.12	0.09	0.12
Actual/pre-event BW (%)	97±0.8	96±0.6	0.49	0.54
**Blood**				
ESR 1^st^ hr (mm)	30.45±25.11	58.75±35.76	<0.0001	0.0002
Haemoglobin (g/dl)	13.27±1.86	12.14±1.94	0.006	0.031
Blood urea (mg/dl)	39.74±18.04	42.57±28.98	0.53	0.56
Serum creatinine (mg/dl)	0.97±0.28	0.98±0.29	0.83	0.83
Plasma glucose (mg/dl)	108.45±36.40	115.04±36.82	0.40	0.47
α_1_ globulin (g/dl)	0.21±0.04	0.24±0.06	0.008	0.033
Fibrinogen (mg/dl)	376.2±89.3	437.1±110.8	0.005	0.031
Serum albumin (g/dl)	3.41±0.53	3.08±0.57	0.005	0.031
Serum prealbumin (mg/dl)	21.57±5.60	19.41±6.16	0.09	0.12
Serum transferrin (mg/dl)	209.56±50.04	188.26±38.73	0.044	0.09
Serum C-reactive protein (CRP) (mg/dl)	1.12±2.35	2.25±2.58	0.031	0.08
White cell counts (mm^3^)	6431.55±1915.17	7492.86±1908.19	0.011	0.038
Neutrophils (%)	58.95±10.37	63.71±9.04	0.030	0.08
Lymphocytes (%)	28.47±9.68	24.30±8.39	0.041	0.09
**Function**				
FIM (score)	61.96±27.37	51.04±29.10	0.069	0.11
**Daily nutritional intake**				
Energy				
Kcal/d	1670±177	1460±195		
Kcal/Kg	22.9±3.51	20.3±2.69	0.08	0.11
Proteins				
g/d	70.5±12.5	61.9±10.9		
g/Kg	0.97±0.18	0.86±0.15	0.09	0.12
Lipids				
g/d	60.3±11.6	44.5±8.6		
g/Kg	0.83±0.16	0.62±0.12	0.1	0.12
Carbohydrates				
g/d	219.3±48	192.7±42.2		
g/Kg	3.07±0.66	2.68±0.55	0.07	0.11

Data are expressed as mean ±standard deviation (SD)

p-values are from the unpaired Student t-test; FDR-adjusted p value: p values for false discovery rate controlled at 5% using the Benjamini-Hochberg method

FIM = functional independence measure; ESR: erythrocyte sedimentation rate

In summary, the patients with low Trp and those with low Gln compared to their respective counterparts shared low circulating Hb, Alb, Transferrin and higher inflammation. In addition, we observed that impaired nutrition and neurocognitive dysfunction were only present in subjects with low Trp, whereas an unbalanced immune response consisting of high innate immune activity and a low adaptive response characterised stroke subjects with low Gln.

## Discussion

The study shows that fasting stroke patients may have an increased amount of plasma AAs whose concentrations (for the majority) were correlated with circulating Alb and Hb levels. The study also found that plasma Trp and Gln were the best positive predictors of Alb and Hb, respectively, but rejects our original hypothesis that isoleucine could predict Hb.

### Patient characteristics

Increased plasma levels of 71.4% of AAs in stroke patients may seem paradoxical given their persisting inflammation and inadequate nutritional intakes [37,38], and even more so when considering the high EAA concentrations, including BCAAs, which is the main fuel for skeletal muscles. However, plasma AA may actually reflect an increased AA release from hypercatabolic skeletal muscle tissue. The following considerations may suggest the presence of muscle hypercatabolism. Firstly, loss of body weight, insufficient nutrient intakes, inflammation and hypoalbuminemia are all highly suggestive of general body hypercatabolism. Secondly, the patients’ high levels of the AAs that are not metabolised by muscle, such as phenylalanine, tyrosine, lysine and methionine, are indicative of a state of muscle hypercatabolism. A previous investigation showed that the ipsilateral arm (= unaffected) in stroke patients had a net release of phenyalanine [9]. Thirdly, high serum CRP suggests increased Interleukine 6 production [44], which promotes muscle protein breakdown by several mechanisms including both inhibition of muscle protein synthesis, repair, contractility and function [45], and stimulation of the hypothalamus-pitituary-cortical axis leading to increased cortisol production. Lastly, high EAA/TAA and BCAA/TAA ratios observed in the study patients were previously reported in cancer individuals with cachexia [46,47].

The association of hypoalbuminemia and high plasma AAs, as observed in the current study, is a different condition from that of starved subjects in whom serum Alb is spared due to increased AA release from skeletal muscle [28]. This suggests that inflammation in fasting stroke patients may divert the majority of circulating AAs from Alb synthesis to the synthesis of positive proteins of acute-phase response [48] and other body districts, especially immune cells [49].

Low plasma levels of aspartic acid, glutamic acid and asparagine probably reflect their body overconsumption as intermediates of cell tricarboxylic chain acid cycle. Inflammation and oxidative stress [50,51] may be contributing factors to low taurine concentrations.

Inflammation, accelerated Alb distribution from the intravascular space [52] and increased degradation following acute metabolic stress after the vascular event can explain the low level of circulating Alb. In experimental animals, a single injection of the cytokine TNF alfa decreased serum Alb concentrations [53]. In a context of metabolic perturbation, the inadequacy of protein-energy intakes contributes to patient hypoalbuminemia.

The stratification of the patients in terms of Trp levels highlighted the negative effects of inflammation on patient nutritional-, metabolic- and functional variables. The subjects with low Trp, compared to those with normal Trp, had a higher inflammation rate associated with lower serum levels of negative proteins of the acute phase response, lower protein-energy intakes, higher loss of body weight and more severe physical disability. Given that Trp is an EAA, its reduced levels were due to an increased body overconsumption/protein intake ratio.

Hypoalbuminemia is an important risk factor for poorer rehabilitative outcomes given that it is associated with increased serum cortical levels, which potentially stimulate muscle breakdown, particularly in immobilised individuals [54], like the study patients. On the contrary, normal Alb levels favour patient autonomy recovery not only because the protein may enhance neurocognitive recovery [16] but also because it reduces the risk of sarcopenia.

### Correlations between plasma AAs and circulating Alb

The study found that the majority of plasma AAs were associated with circulating Alb levels and that Trp and, to a lesser extent, His were predictors of the circulating protein.

The observational nature of the study and correlations do not allow us to infer a cause-effect relationship between AAs and Alb. Yet we postulate that the levels of plasma AAs, primarily Trp and His, could contribute to maintaining Alb synthesis during night fasting. The following factors may support this hypothesis. Firstly, during fasting, Alb synthesis [33] is not abolished, although it occurs at a reduced rate [55–57]. Secondly, Trp availability is essential for Alb synthesis [35]. The biosynthesis of Alb in fasting rabbits [35] was rapidly stimulated by adding Trp to perfusate. In this experiment, the Trp-associated Alb synthesis rate (+138%) was impressive when compared to isoleucine-associated Alb synthesis (+89%), and even more so when compared to a mixture of AAs that did not contain Trp and isoleucine (no increase of Alb synthesis). On the other hand, dietary Trp deficiency in rats inhibits liver synthesis [24]. It is worth noting that the correlation of Trp and Alb remained significant within normal ranges of Trp levels, thus indicating their continuous close connection.

The rate of Trp/Alb associations was higher when Alb was expressed as absolute values rather than when it was normalised for the degree of inflammation (Alb/CRP). This may indicate that the link is even more significant under inflammatory stress that in non-inflammatory conditions. This would confirm that during acute metabolic stress, Alb synthesis increased even though it was at a lower rate than that of the acute phase response [58].

Our hypothesis on Trp contributing to Alb synthesis during nocturnal hours is in contrast to the general belief [22] that during the post absorptive period, Alb catabolism may release EAAs for muscle and other tissue proteins. Bearing in mind that we are in the theoretical field, we think that our hypothesis may be more biologically plausible. Under normal conditions, the increase in Trp levels depends on a decrease in serum Alb and/or an increase in Non-Esterified Fatty Acids (NEFAs) [59], which displace Trp from Alb [29]. In the current study, this was not the case as both low and normal Trp were associated with normal and low Alb.

In addition, if plasma Trp levels in the study were due to Alb dismission of the AA, a negative correlation between the two substances would have been found.

A possible Trp displacement from Alb by NEFAs was unlikely, given that plasma triglyceride and glucose concentrations were normal, thus excluding increases in plasma NEFA levels.

Plasma HIS was shown to be predictive of circulating Alb. Again, there seems to be a cause-effect relationship, given that in the case of a His-deficient diet, whole-body protein turnover decelerates [60] and circulating Alb decreases [61]. This suggests that endogenous [62] synthesis and body store (Hb, carnosine) are not enough to preserve the levels of visceral proteins [61].

The EAA phenyalanine was shown to be a strong negative predictor of Alb. Given that plasma phenyalanine indicates muscle hypercatabolism, the study suggests that muscle hypercatabolism may predict the occurrence of reduced Alb mass. This is in line with the positive relationship between Alb and muscle mass observed in elderly patients, like the study patients [63].

### Correlations between plasma AAs and circulating Hb

More than 77% of plasma AAs in post absorptive stroke patients were positively associated with circulating Hb levels. Plasma Gln levels were positive predictors of Hb, whereas asparagine and glycin were negative predictors.

The provision of AAs to bone marrow is essential for haemopoiesis. Optimal AA availability influences haemopoiesis both directly by promoting protein synthesis in reticulocytes, which are supplied with haeme [34,64,65], and indirectly by influencing erythropoietin production [30]. In anaemic rats, haemopoiesis was significantly stimulated by isoleucine [36] and this AA was essential for the recovery of Hb and erythrocytes even though Hb does not contain isoleucine. These metabolic activities of AAs can explain the links between AAs and circulating Hb found in the present study.

Trp also contributed to haemopoiesis in anaemic rats [36] The positive correlation between plasma His and circulating Hb indirectly confirms previous investigations that documented the importance of His for haemopoiesis. Indeed, His-deficient regimen brings about decreases in Hb concentrations haematocrit, red blood cells [60] by causing the reduction in Hb synthesis [66] and/or increase in Hb degradation [67].

The rate of correlations between EAAs and Hb was higher than the rate of correlations between EAAs and Alb. The huge processes of protein synthesis in bone marrow that are necessary for the formation, replication and differentiation of progenitor cells of erythrocytes as well as the haemoglobinisation of red cells are likely to account for the great need for EAAs.

The results reject our hypothesis of isoleucine as a predictor of Hb. The profound differences between previous experimental studies and the current investigation carried out in patients with metabolic alterations may explain the discrepancy. Glutamine, on the other hand, was shown to be the best positive predictor for circulating Hb. The predictive value of Gln may rely on its vast array of metabolic activities, including the nitrogen provision for the other AAs [68], the essential role in gluconeogenesis providing energy [68], the correction of intracellular redox by enhancing the NAD/NADH ratios [69–71] which are particularly relevant to haemopoiesis.

A mixture of Gln + BCAA + arginine increased Hb, erythrocyte count, haematocrit and serum iron [72]. Gln can protect mammalian erythrocytes against oxidative stress and apoptosis [73]. The metabolites of Gln such as alanine, citrulline and proline effectively protect erythrocytes by suppressing the generation of free radicals, release of cytokine C, activation of caspase-3, caspase-8 and caspase-9. Gln normalises red cell morphology and reduces red cell adhesion in subjects with sickle cell disease [74]. Low erythrocytes contents in Gln and glutathione contribute to an altered redox state of the cell and to haemolysis [75].

Low plasma Gln in stroke subjects (22.4%) was likely due to stroke-primed metabolic stress and not to insufficient protein intakes, given that patients with low and normal Gln levels were similar in daily protein intakes.

The study suggests that in the case of high plasma asparagine and/or glycin, the Hb levels could be impaired. The negative roles of these AAs on Hb are not clear. An excess of asparagine could react with glucose and fructose, leading to Hb glycation end products and post-translational modification of Hb [76]. In the present study, however, asparagine levels were lower in stroke subjects than in controls. This may be due to body overconsumption and/or reduced asparagine synthase expression, the enzyme responsible for the biosynthesis of asparagine from aspartate and glutamine [77].

With respect to glycin, it is of interest that while glutamine increases protein synthesis, an isonitrogenous amount of glycine decreases protein turnover [78]. This may contribute to explaining the inverse correlation between glycin and Hb. The high value of plasma glycin found in stroke subjects was probably due to muscle release following protein overdegradation [9].

Inflammation was likely to have been the main reason for the anaemia that was encountered in one third of the study patients. Indeed, TNF alfa and IL-6 can suppress erythropoiesis and the action of erythropoietin [79], and impair the mobilisation of iron from macrophages [80]. Deficiencies in dietary intakes of iron, vitamin B and phosphorus, as observed in subjects with low Gln, could have contributed to anaemia. Even though it was not measured in the study, low serum vitamin D may contribute to anaemia, particularly during inflammation [81,82].

It is of interest that in the study, low plasma Trp was associated with patient nutrition decline, whereas low Gln was linked to immunological alterations. This confirms that Trp is an important anabolic AA [35,36] and that Gln is essential to immune cell functions [83,84].

## Conclusion

The study shows that subacute stroke subjects in a post absorptive state may have altered levels of plasma amino acids, the majority of which are positively associated with circulating Alb and Hb levels. Plasma Trp and glutamine were the best positive amino acid predictors of circulating Alb and Hb, respectively. isoleucine levels are not predictors of circulating Hb.

## Future studies

The current study is a hypothesis-generating work.

A well-planned investigation could test the hypothesis of improving circulating Alb and Hb in stroke subjects during nocturnal hours by selectively elevating the plasma concentrations of Trp, His and Gln concentrations. This may be of particular importance for stroke individuals with hypoalbuminemia and/or anaemia, particularly within 30 days of the acute event, for at least three reasons. Firstly, during the time in which the majority of neurocognitive recovery occurs, elevating circulating Alb and Hb may be an additive value for the brain dysfunction recovery [10,16]. Indeed, adequate nutrition support takes weeks or months to elevate serum Alb concentration [18]. Secondly, frequently stroke subjects with an oral diet have deficient nutrition intakes [37,38], leading to a reduction of meal-stimulated Alb/Hb synthesis. Thirdly, Trp and His are precursors of the brain neurotransmitters serotonin and histamine, respectively, which are important for motor, cognitive and mood recoveries in stroke patients.

Given the clinical importance of normal Alb and Hb values, there is a need to investigate the relationships between the proteins and circulating amino acids in diseases other than stroke.

## Limits of the study

In this study, we excluded patients with diabetes, chronic kidney disease, chronic obstructive pulmonary disease, hepatic cirrhosis, cancer and all diseases associated with important plasma AA abnormalities. Given the high prevalence of some of these diseases, future studies should address the relationship between plasma AAs and Alb in a context of pre-stroke plasma AA alterations.

Patients ingestions of the AAs were not calculated because not all the enteral formulas that were used in our study reported the AA composition.

The study was carried out in a single Rehabilitation Centre. A polycentric investigation may provide more information, particularly in relation to the setting of provenience of the patients (neuro intensive care unit, stroke unit, neurological departments). Moreover, this led to a relatively small sample size of the healthy control group since only a few subjects underwent the complete set of assays defined in our protocol. However, only type II error rate should be affected, with a potential reduction of statistical power.

Evaluating patients’ body composition would have strengthened the discussion, especially in relation to plasma AAs.

## Suggestions for clinical practice

Even though it is of observational nature, the study provides some information which may improve nutritional/metabolic treatments of subacute stroke subjects. Keeping in mind that the optimisation of the nutrition of stroke patients within 30 days from the acute event is of paramount importance for neurocognitive recovery, it is necessary to calculate the adequacy of nutrient intakes in all stroke subjects, particularly in self-feeding subjects. Moreover, one should consider that actual nutritional adequacy might not be synonymous with normal plasma AAs, particularly in inflamed subjects. On the basis of this study and given that plasma AAs are not routinely measured, it may be convenient to suspect low Trp in stroke individuals with inflammation and >5% loss of pre-event body weight.

The corrections of inadequate nutrition are relevant to subjects suffering from stroke, not only to enhance neurocognitive retrieval early during the post-acute period, but also to continuously improve overtime rehabilitation outcomes. Indeed, these patients 6 months after a stroke may have substantial energy and protein deficits [38].

## Supporting information

S1 File Minimal dataset. (PDF)
