# Supplementary material for: The relationship between plasma amino acids and circulating albumin and haemoglobin in postabsorptive stroke patients
Source: PLoS One. 2019 Aug 14;14(8):e0219756. doi: 10.1371/journal.pone.0219756 (PMC6693779; doi:10.1371/journal.pone.0219756)
Supplement: S1 File — (PDF) [file pone.0219756.s001.pdf]

id_code	Grp	ASP_T0	Glu_T0	HIS_T0	ASN_T0	SER_T0	GLN_T0	3MeH_T0	ARG_T0	Citr_T0
1	0	15	50	78	49	108	717	6,5	71	53
2	0	11	61	53	49	88	515	2	59	25
3	0	13	73	77	55	82	678	3	51	27
4	0	11	68	62	48	114	533	3	78	33
5	0	13	69	84	62	114	612	6,5	65	33
6	0	13	112	80	69	176	695	4,7	79	24
7	0	9	70	64	39	117	504	3,2	26	27
8	0	7	33	74	54	103	500	3,2	94	35
9	0	10	33	62	38	61	543	4	57	37
10	0	11	69	78	45	102	647	4,8	48	28
11	0	9	122	59	47	155	501	2	44	23
12	0	13	53	56	56	146	566	5,9	96	28
13	0	11	47	47	52	104	620	2,4	55	24
14	0	13	34	40	41	123	545	3,1	56	22
15	0	11	70	64	48	105	537	6,2	65	49
16	0	9	87	85	51	119	691	3,7	74	42
17	0	15	68	69	35	110	444	3,4	43	36
18	0	15	38	77	46	99	544	4,3	43	32
19	0	21	34	55	41	94	504	4,1	39	44
20	0	13	66	84	63	173	662	3	72	52
21	0	12	78	93	65	160	656	5,8	83	38
22	0	13	66	86	52	103	501	3,6	66	34
23	0	10	62	41	38	68	513	2	33	27
24	0	10	69	75	42	110	636	3,8	40	23
25	0	11	96	91	52	136	628	3,7	52	32
26	0	11	66	80	53	103	575	10	74	52
27	0	13	80	125	125	243	1082	4,1	131	35
28	0	9	41	56	51	73	456	3,1	36	22
29	0	13	66	73	42	92	530	5,7	44	54
30	0	15	52	97	67	156	805	3,5	78	56
31	0	10	87	74	43	140	583	3,9	72	32
32	0	8	77	77	51	113	611	4,2	58	38
33	0	11	68	62	43	64	501	4,7	34	34
34	0	12	84	90	62	101	774	3,9	36	41
35	0	10	90	73	44	113	552	4,1	71	30
36	0	12	172	56	36	124	419	3,3	53	22
37	0	11	64	84	53	85	500	5,1	63	34
38	0	12	63	77	50	104	635	4,3	46	29
39	0	13	63	90	56	175	725	3,5	92	40
40	0	9	67	80	46	83	514	3,8	57	24
41	0	11	50	76	63	107	796	7,1	96	44
42	0	13	87	101	57	122	651	3,9	59	33
43	0	7	90	50	44	101	351	3,4	58	14
44	0	9	71	57	33	90	448	3,7	50	20
45	0	7	36	38	32	58	289	3,7	30	22
46	0	13	137	84	44	81	498	4,8	56	36
47	0	11	111	45	40	134	461	3,7	33	8
48	0	13	73	74	49	98	645	5	71	47
49	0	12	95	104	55	122	626	4,4	53	35

50	0	15	79	81	49	187	673	6,6	66	40
51	0	10	66	76	35	81	575	5,8	26	33
52	0	10	92	91	52	93	524	2,6	94	45
53	0	12	71	72	36	87	476	3,9	45	26
54	0	13	101	51	41	138	620	3,5	47	23
55	0	15	88	79	52	126	583	4,3	36	50
56	0	11	73	59	41	125	606	3,9	65	38
57	0	9	60	74	43	111	536	4,1	50	32
58	0	9	70	73	39	95	515	5,4	49	38
59	0	13	81	86	46	129	556	4,1	58	33
60	0	12	51	92	63	133	543	4,8	110	45
61	0	13	113	73	64	161	887	4,2	97	30
62	0	11	48	69	51	98	637	4,2	84	35
63	0	13	71	107	62	118	658	4,6	66	38
64	0	10	91	93	58	166	899	3,4	58	57
65	0	12	59	91	62	105	677	3,8	77	27
66	0	11	80	106	70	138	554	4	98	36
67	0	10	59	68	50	115	670	4	59	31
68	0	9	55	87	62	114	746	2	94	41
69	0	13	78	61	40	77	671	5	64	33
70	0	10	65	68	51	118	754	3,6	73	41
71	0	12	55	76	52	120	554	5,3	78	34
72	0	10	86	97	53	111	865	4	45	46
73	0	11	91	67	50	118	519	3,7	79	18
74	0	11	83	81	44	148	535	2,7	53	37
75	0	13	55	75	44	131	596	3,4	45	31
76	0	11	55	45	52	80	542	4,7	49	22
77	0	13	41	70	50	89	563	3,4	61	28
78	0	15	61	78	67	95	713	5,9	44	39
79	0	11	72	87	68	147	670	4,5	57	31
80	0	12	72	75	47	113	540	3,6	74	23
81	0	11	77	81	51	116	578	4,3	41	32
82	0	11	128	60	37	72	473	4,6	43	36
83	0	10	49	55	47	157	555	4,3	46	25
84	0	7	68	54	39	76	451	4	33	32
85	0	13	76	75	49	102	487	3,6	65	32
86	0	14	71	84	53	139	593	3,6	78	28
87	0	12	61	65	53	118	555	3,2	4	27
88	0	10	51	67	80	99	664	3,6	90	37
89	0	8	56	57	34	106	402	3,6	45	15
90	0	13	63	77	54	122	658	3,9	58	36
91	0	11	60	77	55	146	667	4,3	49	46
92	0	12	75	53	44	111	570	3,6	59	19
93	0	12	81	78	64	180	665	3,6	46	30
94	0	12	104	76	56	112	510	3,3	54	31
95	0	9	56	47	48	79	358	3,9	63	22
96	0	11	73	69	36	83	457	2,9	68	17
97	0	11	112	62	52	120	800	3,4	59	32
98	0	9	44	66	43	119	530	4,1	83	40
99	0	13	46	85	65	101	636	4,6	58	47

100	0	11	82	64	48	128	523	4,2	50	43
101	0	14	102	74	43	99	481	5,3	43	46
102	0	12	68	43	38	78	374	6,5	70	33
103	0	16	54	73	54	135	483	4,1	63	44
104	0	14	59	78	52	141	582	4	50	42
105	0	8	58	60	38	97	395	4,8	45	21
106	0	10	130	77	45	103	561	4	49	42
107	0	7	65	53	50	140	543	3,8	45	17
108	0	14	53	69	46	95	627	5,1	50	34
109	0	15	60	80	47	85	466	3,7	80	38
110	0	10	41	63	41	91	674	3,8	53	24
111	0	8	56	52	43	102	450	3,3	54	26
112	0	8	64	78	42	104	597	4	58	32
113	0	13	74	88	51	121	516	4,2	47	46
114	0	11	148	57	47	116	531	3,1	65	18
115	0	7	80	48	36	76	500	4,5	34	17
116	0	8	69	38	27	72	389	3,6	38	20
117	0	8	71	73	44	115	495	3,5	40	39
118	0	9	108	72	40	93	482	3	70	22
119	0	12	77	67	41	143	512	4,1	61	34
120	0	9	66	85	45	127	578	3,7	83	34
121	0	13	62	75	45	63	563	4,6	54	36
122	0	13	69	67	52	95	502	4,8	81	34
123	0	13	82	68	34	112	576	4,8	41	19
124	0	14	46	76	49	118	582	5,6	59	34
125	0	13	37	71	15	156	489	4,3	43	14
C1	1	120	195	59	62	91	451		50	20
C2	1	125	210	61	59	95	471		66	26
C3	1	111	199	57	59	82	475		59	20
C4	1	120	215	63	64	89	450		71	30
C5	1	103	189	64	59	90	456		50	24
C6	1	101	185	51	62	87	490		64	29
C7	1	60	191	59	63	84	470		60	25
C8	1	105	205	50	62	90	456		54	23
C9	1	12	171	37	58	37	89		60	
C10	1	7	90	38	36	33	77		46	
C11	1	10	145	35	58	49	113		54	
C12	1	4	57	60	29	46	433		63	
C13	1	7	108	68	36	46	80		83	
C14	1	7	50	63	44	44	417		71	
C15	1	12	159	40	67	71	65		57	

GLY_T0	THR_T0	ALA_T0	TAU_T0	TYR_T0	g_ambutir_T0	VAL_T0	MET_T0	TRP_T0	PHE_T0	LEU_T0
395	161	384	80	54	5	225	24	41	53	104
231	126	318	77	69	9	226	25	39	76	124
369	190	438	76	76	12	277	37	42	63	143
202	158	385	74	67	15	239	22	43	64	134
304	179	483	97	78	8	239	31	62	62	152
259	180	337	73	49	5	161	26	50	39	95
257	125	419	54	48	14	234	16	33	48	121
219	115	245	74	54	34	308	37	37	65	170
241	71	190	78	37	10	172	21	31	55	109
251	127	395	71	55	28	314	24	46	53	171
216	99	342	38	63	29	230	28	52	43	139
252	194	241	91	64	9	201	39	39	60	132
432	112	363	74	56	16	127	27	29	47	75
279	102	226	39	46	30	147	20	26	46	80
225	129	294	84	67	41	355	38	46	77	209
241	118	315	156	61	25	323	27	46	54	157
289	112	312	89	42	17	203	23	30	46	117
205	118	311	46	52	21	236	26	34	58	125
251	126	309	81	51	13	161	19	35	55	94
285	158	280	63	81	34	267	30	48	70	169
280	155	447	117	130	48	320	42	53	77	179
203	116	358	75	52	12	267	42	45	142	135
279	60	182	142	57	3	149	14	24	60	63
256	138	474	60	57	29	263	30	44	51	132
304	191	462	103	80	25	272	35	49	63	147
237	100	359	156	63	27	248	31	45	78	149
329	248	674	101	138	46	563	33	115	140	342
189	94	314	86	78	12	208	21	38	68	105
273	128	273	73	52	28	288	30	39	56	153
269	286	369	66	112	44	492	66	47	66	147
212	155	255	133	45	13	240	40	47	50	131
184	135	348	97	86	49	424	47	56	70	207
205	133	247	82	42	21	222	25	31	79	124
227	160	395	65	69	27	269	36	43	55	142
254	96	215	60	63	6	382	42	28	55	92
320	101	213	194	57	5	166	26	28	59	98
280	127	436	139	71	12	212	24	48	68	122
266	148	314	84	66	10	268	28	34	52	142
343	177	348	104	52	9	220	25	37	46	101
198	137	432	85	76	13	294	29	47	64	157
454	238	371	80	56	23	125	27	29	64	65
192	126	439	79	80	28	274	31	53	64	152
254	137	220	84	84	27	190	28	44	57	112
299	99	265	104	50	24	205	25	35	49	100
146	56	183	93	51	11	207	18	39	64	120
244	110	324	133	91	13	307	46	51	63	155
254	75	298	89	44	11	194	26	26	40	93
342	179	374	142	52	10	139	20	36	73	100
276	188	596	110	60	12	178	24	48	62	151

386	179	362	156	39	17	174	16	40	61	137
277	77	331	104	48	7	115	13	46	63	114
265	222	534	68	90	17	277	50	60	71	153
202	88	279	100	85	11	258	20	49	57	150
534	148	372	97	61	32	198	25	46	51	98
286	164	276	83	58	11	213	26	46	51	126
276	138	294	82	47	27	223	23	38	47	119
193	103	327	72	80	29	344	32	47	90	190
286	138	310	86	64	12	245	26	35	78	114
209	129	402	96	73	22	272	32	41	52	147
435	175	348	86	87	12	228	34	52	67	121
337	172	371	94	82	15	240	29	52	60	147
277	138	357	78	62	12	206	33	33	55	115
248	226	523	148	71	23	421	42	51	65	197
390	152	435	109	70	21	328	30	51	61	188
263	157	366	145	70	16	305	48	53	64	176
325	172	451	86	103	21	329	45	59	59	177
295	110	210	100	50	11	155	18	44	49	92
251	155	379	79	82	6	260	28	54	69	154
243	107	329	76	54	15	210	30	31	45	94
537	160	335	58	77	11	265	25	19	44	123
320	129	372	128	56	18	365	39	39	71	193
309	195	326	125	63	14	283	28	34	49	137
202	164	419	77	68	21	294	25	50	70	191
257	147	280	96	60	11	226	27	40	43	118
246	182	343	75	54	23	235	30	36	51	120
217	131	418	71	86	12	262	32	23	57	132
274	184	380	69	79	8	258	37	55	58	125
285	90	441	110	77	9	220	28	44	91	137
250	219	373	110	69	22	354	31	59	70	199
177	135	262	90	67	14	299	25	48	61	165
186	136	387	97	64	16	361	28	44	63	223
204	70	427	85	63	45	263	20	43	61	139
328	106	207	102	46	13	184	18	30	50	93
150	76	209	77	74	13	204	22	34	63	120
256	170	484	126	91	39	243	28	48	66	129
293	156	339	103	71	29	267	32	64	58	159
247	167	366	110	70	17	201	26	46	36	112
284	178	447	155	66	24	170	27	38	60	97
197	82	269	98	37	22	189	17	38	50	120
338	196	360	67	74	28	252	28	28	57	128
286	174	277	138	51	22	213	25	38	55	104
316	139	465	156	46	15	182	24	41	45	103
324	224	258	131	46	39	236	22	36	47	132
275	155	293	118	92	32	262	30	51	82	166
211	106	215	65	78	30	215	34	37	86	125
192	82	325	81	52	14	192	23	48	48	110
235	128	317	102	67	34	221	26	36	44	134
253	89	290	119	47	25	199	19	51	52	106
299	139	491	148	73	20	248	32	43	62	157

242	114	325	87	78	34	248	24	41	63	140
206	114	237	99	53	31	248	19	39	57	141
137	89	223	144	66	20	277	28	26	76	146
391	163	385	68	85	18	232	33	36	59	124
369	182	352	69	64	22	209	29	49	54	115
236	85	238	68	55	20	261	23	36	76	153
231	110	340	79	69	21	217	26	43	54	123
340	153	153	99	49	56	216	22	28	64	137
294	150	336	57	45	44	317	26	28		290
364	143	438	183	59	16	280	29	58	73	159
312	98	264	60	44	7	155	27	33	43	83
244	115	261	114	65	14	204	30	42	88	108
205	114	279	53	53	25	275	25	25	59	160
274	189	376	99	62	47	335	31	53	58	162
221	113	404	117	103	22	242	35	51	76	135
182	77	270	109	71	23	237	22	40	60	128
259	71	225	139	62	15	173	20	34	69	74
241	115	261	70	52	24	289	28	44	59	159
256	115	444	90	74	20	253	28	51	58	149
274	157	300	94	65	37	214	21	50	57	112
273	124	386	78	47	7	210	26	39	55	117
260	133	436	110	92	17	264	32	61	73	139
224	132	415	74	67	26	271	30	46	65	146
287	69	543	62	41	41	257	25	17	39	139
210	122	283	69	51	17	189	25	54	48	113
239	146	553	61	39	35	303	50	44	133	125
270	120	301	120	50		135	8,5	40	45	75
261	110	310	131	55		140	10	55	46	80
273	105	315	121	51		138	13	54	44	81
285	121	300	140	65		149	10	56	49	70
264	98	319	109	54		171	10	58	41	82
254	106	325	135	51		162	8,9	52	52	88
284	100	291	185	65		170	12	48	50	75
255	99	340	125	59		149	8,1	50	41	71
180	90	303		46		187	29	41	44	110
64	92	180		26		108	10	24	25	51
170	106	326		50		171	29	40	42	97
138	57	176		38		157	18	35	32	79
125	78	247		35		135	17	24	33	66
76	155	297		54		158	17	32	37	70
227	129	236		42		160	27	41	43	84

ORN_T0	LYS_T0	ILE_T0	album_T0	HB_T0	sex	age	height	weight_T0
94	147	55	4	13,8	M	37	160	66
75	204	64	3,4	14,9	M	63	170	75
82	228	86	3,2	12,3	F	42	160	57
77	231	70	3,4	16,3	M	46	170	80
52	256	73	4,5	14,4	M	63	174	90
51	216	46	4,2	14,1	M	39	182	80
100	195	65	3,6	13,9	M	54	160	70
73	265	86	3	13,3	M	81	160	48
	129	57	3,5	12,3	M	80	155	55
100	239	87	4	14,9	M	63	178	75
	222	75	3,7	14,8	M	49	172	90
53	236	81	3,2	12,4	M	80	160	55
74	199	56	2,2	10,3	F	77	160	80
70	176	61	2,8	10,8	F	71	162	82
106	291	107	2,6	12,2	M	78	180	83
54	199	70	3,5	15,2	F	63	155	56
87	146	56	3,1	14	F	88	160	74
86	221	62	3,2	12,4	M	62	170	74
88	225	40	2,7	13,2	F	89	150	45
110	247	81	3,6	16,3	M	64	170	65
119	261	94	4,2	12,5	M	75	165	74
119	193	73	4	14,7	M	73	165	97
59	128	25	2,4	10,3	M	66	166	58
75	213	82	3,7	14,2	M	78	175	91
102	218	76	4	15,2	M	64	182	88
105	269	59	3,6	12,7	F	84	150	48
77	453	143	4	14	M	62	170	65
75	141	58	2,6	12,3	F	74	170	76
95	183	92	3,3	12,5	M	69	165	82
33	84	76	3,3	10,9	F	68	160	79
83	237	70	4,3	14,9	M	57	162	62
160	244	105	4	17	M	60	171	87
120	199	62	3,5	13,7	F	75	155	45
162	217	76	3,2	15,7	M	67	182	76
113	116	42	2,3	10,4	F	75	154	53
65	175	56	2,1	11,3	M	65	160	49
114	279	57	3,3	13,2	F	78	150	70
122	215	74	3,2	13,6	M	74	175	80
105	74	50	4	13,5	M	60	174	65
96	291	83	3,4	13,6	F	74	160	82
50	203	40	2,5	8,9	F	74	160	56
86	268	76	3,4	15,3	M	63	164	84
70	263	64	2,9	10,4	M	40	185	85
52	113	60	3,3	10	F	50	160	79
39	161	48	3	9,9	F	83	160	60
110	237	65	3,9	15,6	M	64	175	84
62	99	58	2,4	12,2	F	59	170	103
123	233	50	2,6	11,3	F	95	160	63
116	252	78	3,2	12,9	F	48	155	74

132	234	65	3,5	13,6	F	44	171	115
216	192	38	2,5	11,3	M	63	172	63
131	295	102	3,4	12,8	F	49	165	62
57	226	68	3,7	15,7	M	52	178	98
62	227	56	3,6	12,4	F	77	165	70
99	320	66	3,7	16,1	M	60	175	90
64	259	58	3,4	11,6	M	64	168	60
143	158	102	2,7	13,7	M	80	167	67
58	226	47	3	11,3	F	76	170	110
86	202	79	4	15,8	M	54	165	76
140	67	70	3,6	8,4	M	77	170	72
95	258	88	3,4	14	M	56	180	65
78	234	70	3,4	12,4	M	80	160	50
82	239	106	3,4	13,7	F	40	166	83
121	255	109	3,8	14,1	M	72	180	85
77	228	82	3,7	14,6	M	44	170	70
88	303	93	4,2	14,7	M	76	166	82
124	170	48	4,3	10,7	F	49	170	64
155	209	75	3,7	15,2	M	74	160	62
72	230	55	3,4	12,3	M	67	180	85
91	234	73	3,2	13	F	63	172	54
138	245	105	3,3	14,8	M	78	165	79
97	244	63	3,5	18	F	75	164	58
77	279	90	3,5	14,1	M	61	175	103
64	180	60	3,8	14,9	M	58	168	57
135	250	63	4,1	13,4	F	63	162	89
47	192	89	1,8	9,8	M	87	160	57
43	139	60	3,9	16,6	M	60	185	84
61	170	68	3,2	12,7	F	82	160	62
87	307	108	3,6	13,8	F	77	165	79
74	233	89	3,9	14,1	M	73	170	84
62	271	112	3,8	13,7	M	77	155	77
101	225	69	4	12	F	70	160	54
55	160	59	2,8	13,8	F	78	160	54
50	149	55	2,9	13	F	79	155	45
81	281	68	3,1	16,2	M	77	180	120
71	258	78	4,3	14,01	M	35	165	73
90	217	60	3,8	9,5	M	27	179	65
49	267	50	2,7	8,7	F	75	160	65
53	144	63	3,8	11,1	M	63	170	67
97	212	72	2,8	12,5	M	75	175	68
60	229	63	3,4	12,2	F	85	150	45
83	213	76	3,6	11,6	M	30	180	74
118	318	77	2,9	13,4	F	58	175	79
107	201	95	2,7	12,3	M	76	160	52
106	208	68	2,4	10,5	M	56	165	76
57	195	55	3,7	11,4	F	16	165	70
99	183	76	3,2	13,7	M	67	178	87
65	212	53	3,6	13,4	M	70	180	75
102	258	87	3,9	15,5	M	73	176	62

114	247	74	3,8	14,8	M	88	165	63
99	218	79	3,3	13,3	F	86	160	56
70	179	88	1,8	8,5	M	61	170	63
119	202	72	3,1	12,7	F	79	165	72
113	212	52	3,4	12,6	F	79	166	60
87	227	79	2,8	8,7	F	82	160	73
124	241	61	3,2	13,2	M	73	168	86
80	221	77	2,6	9,5	M	46	170	60
55	229	74	2,9	12,4	F	66	160	79
66	200	74	3,6	12,2	F	53	158	52
40	183	45	2,7	12,9	M	58	172	63
44	199	57	2,2	11,5	F	56	158	47
115	245	77	3,3	15,7	M	77	178	78
142	339	91	3,6	12,8	F	64	158	67
81	261	83	3	13,4	M	39	180	64
67	200	69	2,7	12,9	M	77	180	105
27	162	43	3,3	9,7	M	74	185	70
108	255	68	3,6	12,6	M	76	160	60
60	367	77	3,7	13,6	M	54	185	99
86	200	57	3,6	12,3	F	86	165	60
96	183	49	4	11,4	M	35	168	85
58	190	67	3,2	13,5	M	75	180	95
72	230	80	3,3	13,9	M	64	168	80
47	188	79	3,1	13	F	52	150	59
77	200	54	4	15,5	M	40	170	80
51	146	105	3	12,1	F	79	163	61
55	115	47						
64	125	51						
56	130	43						
50	120	44						
68	104	40						
52	105	48						
55	110	44						
51	127	40						
	124	57						
	51	28						
	121	56						
	63	43						
	100	40						
	71	35						
	85	46						
